# Neonatal demise from a complex abdominal wall defect in a low-resource setting: A case study on the consequences of a fractured perinatal care cascade

**DOI:** 10.1016/j.radcr.2025.08.098

**Published:** 2025-09-22

**Authors:** Abdirahman Adan Osman, Hoda Mohamed Deria, Abdirahman Mohamed Jama, Abdirizak Osman Hersi, Abdiasis Esse Ahmed, Hassan Elmi Moumin, Abdirahman Omer Ali

**Affiliations:** a School of Medicine, College of Health Science, Amoud University; b Pediatric Department, Al-Rahma Hospital, Borama, Awdal, Somalia

**Keywords:** Abdominal wall defect, Gastroschisis, Omphalocele, Health disparity, Perinatal care, Resource-Limited setting, Health system strengthening

## Abstract

Survival for neonates with abdominal wall defects (AWDs) exceeds 90% in high-income countries, yet mortality remains devastatingly high in many low-resource settings. This profound survival disparity reflects systemic deficiencies in the perinatal care cascade. We present a case of a preterm neonate with a complex AWD to exemplify the catastrophic consequences when this continuum of care is fragmented. A 32-year-old multiparous woman presented at 28 weeks gestation. Prenatal ultrasound identified a live fetus with a large, complex AWD; findings were ambiguous, with features suggestive of both gastroschisis (free-floating bowel) and a ruptured omphalocele (significant liver herniation, suggestion of a partial membrane). After counseling, she was referred for tertiary care, but transfer was not feasible due to systemic barriers. Two weeks later, at 30 weeks gestation, she presented in advanced preterm labor and delivered vaginally. The 1.5 kg male neonate was born without spontaneous cardiorespiratory activity (Apgar scores of 0 at 1 and 5 minutes) and had a large paraumbilical defect with massive evisceration of the liver and intestines. Resuscitation was unsuccessful. This case demonstrates that neonatal mortality from complex congenital anomalies in resource-limited environments is often a consequence of health system failures rather than an inevitability of the pathology itself. The diagnostic ambiguity of the defect was clinically secondary to the critical, sequential breakdowns in the care cascade: the inability to enact a timely transfer to a tertiary center for planned delivery and immediate access to pediatric surgical and neonatal intensive care. This outcome underscores the imperative to strengthen referral pathways, build capacity for specialized perinatal services, and invest in regionalized care to address the stark global inequities in survival for treatable congenital conditions.

## Introduction

Abdominal wall defects (AWDs), primarily gastroschisis and omphalocele, are surgically correctable congenital anomalies. In high-income countries (HICs), a well-established system of care involving prenatal diagnosis, in-utero transfer, planned delivery in a tertiary center, and immediate access to multidisciplinary neonatal surgical and intensive care has yielded survival rates exceeding 90% [1,2]. In stark contrast, mortality in many low- and middle-income countries (LMICs) can be as high as 100%, creating one of the most glaring disparities in global pediatric surgery [3]. This gap is not explained by the severity of the anomaly but by systemic deficiencies across the perinatal care continuum.

The successful management of AWDs is predicated on an intact “perinatal care cascade”: 1) accurate prenatal detection and risk stratification; 2) effective maternal counseling; 3) a functional referral system for transfer to a specialized center; 4) planned, multidisciplinary delivery; and 5) immediate access to neonatal surgery, parenteral nutrition, and advanced life support [4]. A breakdown at any point in this cascade precipitates a precipitous decline in prognosis.

Here, we present the case of a preterm neonate with a complex AWD and immediate postnatal demise. The objective is not to highlight a rare pathological variant but to use this case to exemplify the lethal consequences of a fractured care cascade, underscoring the critical role of robust health systems in determining neonatal outcomes. This report adheres to the CARE guidelines [5].

## Case report

A 32-year-old woman, gravida 5, para 4, with an unremarkable obstetric history, presented to a district-level facility at 28 weeks gestation reporting mild, self-resolved vaginal bleeding and lower abdominal pain. Fetal movements were normal. Maternal vital signs were stable, and abdominal examination revealed a gravid uterus consistent with gestational age. The cervix was closed and uneffaced.

**Fig. 1:**
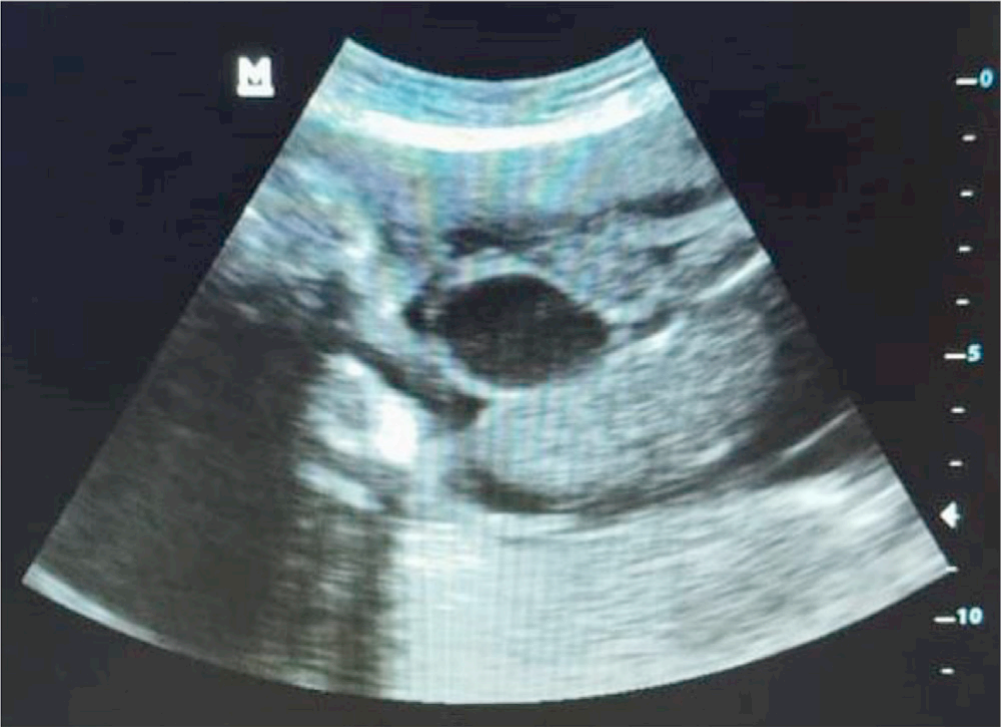
Prenatal ultrasound at 28 weeks gestation (transverse view of fetal abdomen). Extruded abdominal contents are visible anterior to the fetal abdomen, with some areas suggesting partial encasement by thin membranous structures.

**Fig. 2:**
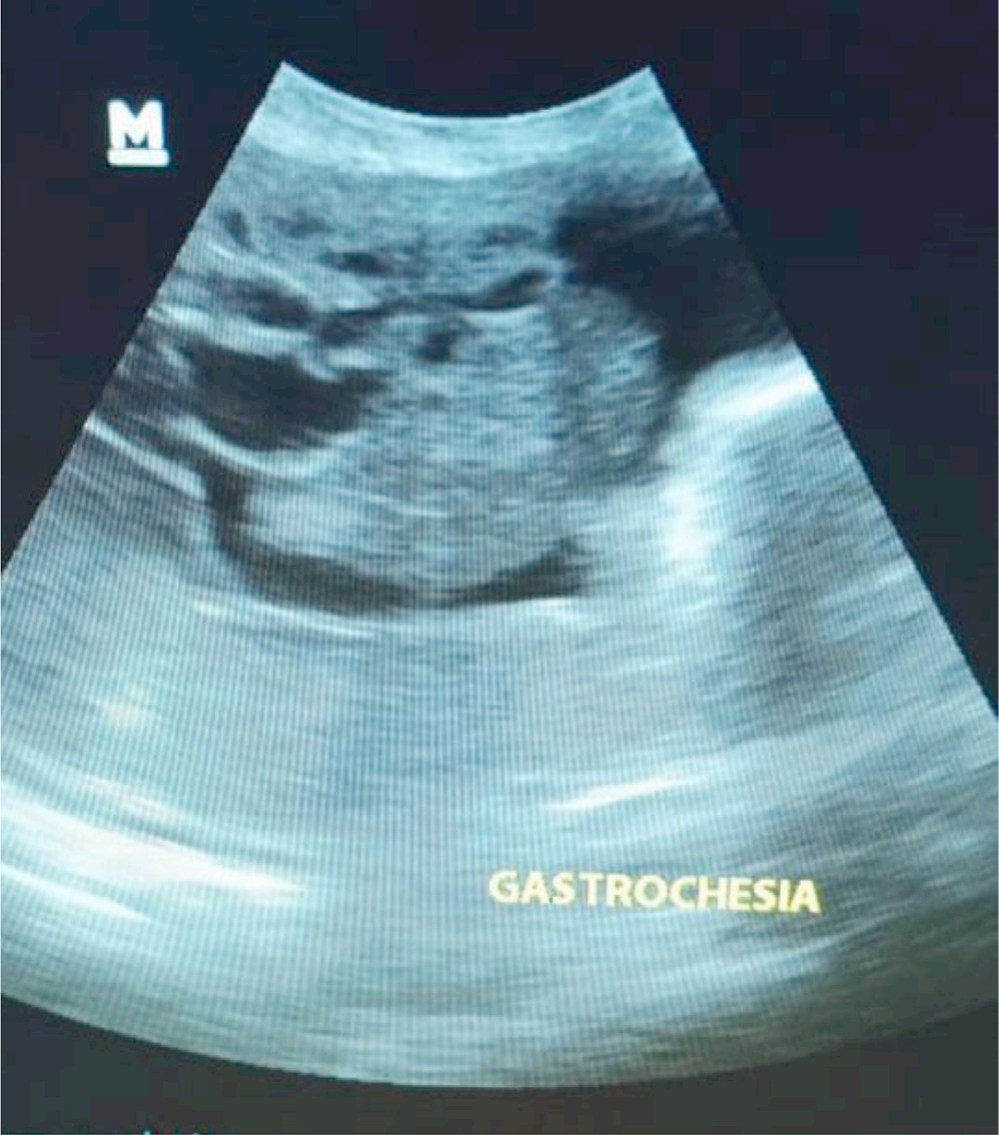
Prenatal ultrasound at 28 weeks gestation (sagittal/oblique view). This image demonstrates free-floating bowel loops (asterisk) external to the fetal abdomen, a characteristic feature supporting gastroschisis.

Urinalysis revealed significant leukocytosis (>25 WBC/hpf), and a urinary tract infection (UTI) was diagnosed. An obstetric ultrasound confirmed a single, live, male fetus with biometry consistent with 28 weeks. A large anterior abdominal wall defect was visualized to the right of the presumed umbilical cord insertion. The sonographic findings were ambiguous; some views demonstrated free-floating bowel loops characteristic of gastroschisis (Fig. 2), while others revealed significant liver herniation and suggested the presence of thin, partially encasing membranous structures (Fig. 1). This combination precluded a definitive prenatal differentiation between a giant gastroschisis and a ruptured or atypical omphalocele.

A working diagnosis of a complex AWD was established. The patient was counseled on the grave prognosis, which was profoundly compounded by the local resource limitations. She was initiated on oral amoxicillin for the UTI and formally referred for specialist care at a tertiary center. However, transfer was not feasible due to insurmountable logistical and systemic barriers.

Two weeks later, at 30 weeks gestation, she presented in active preterm labor. Given the rapid progression and the continued unavailability of transfer, a vaginal delivery was managed locally. Any remaining membranes surrounding the fetal viscera presumably ruptured during the second stage of labor.

**Fig. 3:**
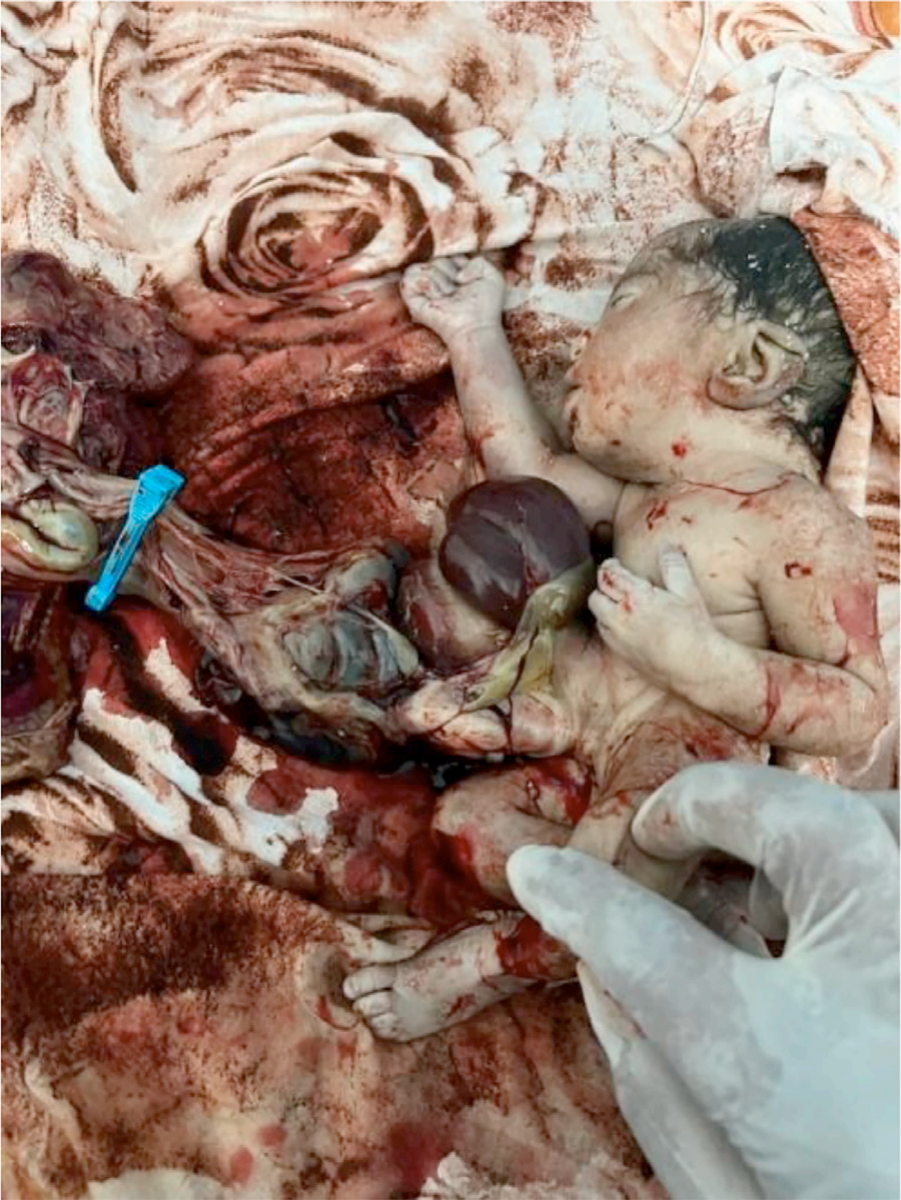
Postnatal appearance of the neonate. A large paraumbilical abdominal wall defect is evident with extensive evisceration of edematous bowel and massive herniation of the liver (L). The umbilical cord (UC) is clamped and visible to the left of the defect.

A 1.5 kg male infant was delivered vaginally. The neonate was born without vital signs—limp, cyanotic, and with no spontaneous cardiorespiratory activity. The Apgar score was 0 at 1 minute and remained 0 at 5 minutes. Postnatal examination confirmed a large (approx. 5-6 cm) paraumbilical defect with extensive evisceration of edematous, congested intestines and a massively herniated liver (Fig. 3). The umbilical cord was inserted to the left of the defect. Clinically, the massive liver herniation strongly suggested the defect was a prenatally ruptured omphalocele. Despite immediate resuscitation efforts, the newborn was pronounced dead 5 minutes after birth. An autopsy was declined by the family.

## Discussion

This case of a lethal abdominal wall defect (AWD) in a preterm neonate underscores the systemic failures that contribute to the significant disparity in neonatal surgical outcomes between high-income countries (HICs) and low- and middle-income countries (LMICs). The mortality rate for AWDs can reach 100% in LMICs, contrasting sharply with survival rates exceeding 90% in HICs, as evidenced by systematic reviews and cohort studies [6,1]. This discrepancy is not merely a function of the anomalies themselves but reflects profound deficiencies in the perinatal care cascade, which encompasses prenatal detection, referral processes, and access to specialized care.

The ambiguity in prenatal ultrasound findings presented a diagnostic challenge, aligning with findings from previous studies that highlight the limitations of prenatal imaging in resource-limited settings [7]. The inability to differentiate between gastroschisis and omphalocele in this case illustrates the complexities faced by healthcare providers in such environments. The subsequent failure to transfer the patient to a tertiary care facility further exacerbated the situation, echoing research that identifies ineffective referral systems as a critical barrier to timely and appropriate care [2].

Contradictory to some reports that suggest improvements in neonatal outcomes can be achieved through enhanced training of local healthcare workers, this case emphasizes that without a functional referral and transfer system, such training may have limited impact. While some studies advocate for localized training initiatives to address knowledge gaps, the systemic barriers observed in this case highlight that training alone is insufficient without corresponding investments in infrastructure and resources [8].

This case contributes to the growing body of evidence advocating for systemic health reforms aimed at strengthening perinatal care in LMICs. It underscores the importance of creating regional centers of excellence equipped to manage high-risk pregnancies and complex congenital anomalies effectively. Previous research has shown that concentrated expertise leads to improved patient outcomes, as seen in various global health initiatives [9].

In the context of the Sustainable Development Goals (SDGs), particularly Goal 3, which aims to ensure healthy lives and promote well-being for all at all ages, this case serves as a poignant reminder of the challenges that remain. The inability to provide adequate care for treatable conditions not only affects neonatal mortality rates but also has broader implications for maternal health and the overall health system. Strengthening healthcare systems to ensure that all children have access to necessary surgical interventions aligns with the SDG commitment to universal health coverage and equitable access to quality essential health services [5].

## Conclusion

This case exemplifies the dire consequences of systemic failures in the perinatal care continuum, emphasizing the need for comprehensive health system strengthening. Future efforts must focus on addressing the multifaceted barriers that prevent timely and effective care for high-risk neonates, thereby contributing to the broader goals of health equity and sustainable development.

## Author contributions

Conceptualization: A.A.O., H.M.D., A.M.J., A.O.H., A.E.A., A.O.A. Data curation: A.A.O., H.M.D., A.M.J. Formal analysis: A.A.O., A.O.A. Investigation: A.A.O., H.M.D., A.M.J., A.O.H. Methodology: A.O.A., A.E.A. Project administration: A.O.A. Resources: A.O.A., A.E.A. Supervision: A.O.A., H.M.D. Validation: A.A.O., A.O.A. Visualization: A.A.O., A.M.J. Writing – original draft: A.A.O., H.M.D. Writing – review & editing: A.A.O., H.M.D., A.O.A. All authors reviewed and approved the final manuscript.

All authors attest that they meet the current ICMJE criteria for Authorship.

## Ethical approval

All procedures involving human participants were performed in compliance with relevant laws and institutional guidelines and were approved by the institutional review board of the College of Health Sciences at Amoud University, the Ministry of Health, and Borama Regional Hospital in the Awdal Region, Somaliland (BRH-260/2024).

## Patient consent

We have obtained written consent from the patient's legal guardian to publish this case report, including any associated images, reports, or clinical data. The guardian has been informed of the publication's purpose, its potential risks and benefits, and the steps taken to ensure patient confidentiality. The patient's identity has been protected, and all identifying information has been removed or altered to maintain anonymity. We acknowledge that this case report holds educational and scientific value, and we grant permission for its publication in a reputable medical journal or platform.

Informed consent was obtained from the patient for publication of this case report and accompanying images. A copy of the written consent is available for review by the Editor-in-Chief of this journal on request.
